# Experimental Autoimmune Encephalomyelitis Is Successfully Controlled by Epicutaneous Administration of MOG Plus Vitamin D Analog

**DOI:** 10.3389/fimmu.2017.01198

**Published:** 2017-10-16

**Authors:** Sofia Fernanda Gonçalves Zorzella-Pezavento, Luiza Ayumi Nishiyama Mimura, Thais Fernanda Campos Fraga-Silva, Larissa Lumi Watanabe Ishikawa, Thais Graziela Donegá França, Alexandrina Sartori

**Affiliations:** ^1^Department of Microbiology and Immunology, Institute of Biosciences, São Paulo State University (UNESP), Botucatu, Brazil

**Keywords:** experimental autoimmune encephalomyelitis, vitamin D analog, MOG, epicutaneous route, regulatory T cell

## Abstract

Multiple sclerosis (MS) is an inflammatory and demyelinating disorder of the central nervous system (CNS). Experimental autoimmune encephalomyelitis (EAE) has been widely employed to evaluate new strategies to control MS, including procedures to induce immunological tolerance. Considering that skin exposure to protein antigens can induce tolerance and that vitamin D analogs conserve immunomodulatory potential and are less toxic, we investigated the efficacy of epicutaneous application of a myelin oligodendrocyte glycoprotein peptide (MOG_35–55_) associated with paricalcitol (PARI) on EAE development. Three and 11 days after EAE induction, C57BL/6 mice were treated with an occlusive patch containing MOG plus PARI. Clinical parameters were daily assessed, whereas immunological and histological evaluations were performed during the acute EAE phase. MOG and MOG + PARI significantly controlled disease development reducing weight loss and clinical score. Moreover, MOG and MOG + PARI reduced the inflammatory process and preserved the myelin sheath in the CNS. High percentages of Foxp3^+^ regulatory T cells (Tregs) and lower MHCII fluorescence intensity in dendritic cells in draining lymph nodes were concomitantly observed. MOG + PARI association was, however, more efficient being able to reduce disease incidence and clinical scores more significantly than MOG or PARI alone. This experimental group also displayed a higher ratio between mRNA expression for Foxp3 and RORc and a higher percentage of Foxp3^+^ cells in the CNS. Modulation of activation markers observed in microglial cells eluted from EAE treated mice were confirmed by *in vitro* studies with the BV-2 microglial cell line. The results show that MOG + PARI association applied by an epicutaneous route controlled EAE development. Protective involved mechanisms include mainly a higher proportion of Tregs and also a direct immunomodulatory effect of PARI on microglial cells.

## Introduction

Multiple sclerosis (MS) is a chronic autoimmune pathology of the central nervous system (CNS) characterized by local inflammatory infiltration, demyelination, and axonal damage, which affects mostly young adults (1). The etiology of MS remains elusive, but it is accepted that self-reactive Th1 and Th17 cells with specificity for myelin sheath antigens play a central role in initiation and perpetuation of CNS inflammation (2, 3). Experimental autoimmune encephalomyelitis (EAE) is an artificially induced disease of the CNS that resembles MS in its clinical, histopathological, and immunological features. In this model, myelin-specific CD4^+^ T cells activated in peripheral lymphoid organs pass through the blood–brain barrier (BBB) and invade the CNS. Recognition of myelin peptides on the surface of local antigen-presenting cells (APCs) leads to re-activation of these CD4^+^ T cells, resulting in pro-inflammatory cytokine production, oligodendrocyte damage, and axonal demyelination (4–6). In addition to myelin and axonal injury directly performed by TCD4^+^ and TCD8^+^ lymphocytes, this disease is also mediated by persistent activation of microglial cells and macrophages, antibody, complement components and also by a plethora of mediators as cytokines, nitric oxide (NO), and metaloproteinases (7–9).

Several disease-modifying therapies (DMTs) were already approved by the US Food and Drug Administration (FDA) for MS treatment. None of these medications determines cure, but they decrease clinical relapses and avoid the appearance of new lesions. IFN-β, glatiramer acetate, and natalizumab are some the most employed DMTs. The immunomodulatory activity of these drugs includes decreased Th1 and Th17 activation, reduced BBB permeability, lower lymphocyte migration to the CNS, and upregulation of IL-10 production (10). Fingolimod was recently approved by the FDA as a therapy for patients with severe MS. This is an oral sphingosine-1 phosphate (S1P) receptor modulator that prevents lymphocyte egression from the lymph nodes and triggers neuroprotective effects through interaction with S1P receptors on neural cells (10, 11). Despite numerous and innovative therapies for MS, none of them is able to completely break neurodegeneration associated with the disease. Severe side effects such as increased susceptibility to infections, depression, thyroid dysfunction, cardiotoxicity, and abnormalities in liver enzymes (10) can be associated with these therapies. By contrast, approaches aiming immunological tolerance induction to myelin would be theoretically more efficient and without major deleterious effects. Intravenous, oral, or transdermal administration of myelin antigens have been described as potentially therapeutic for EAE (12–15). Epicutaneous application of purified self-antigens seems to hold a great promise in therapeutic approaches against autoimmune diseases (16). This procedure leads to development of immune tolerance and has been applied in human trials (17). Epicutaneous immunization with myelin basic protein (MBP) prior to EAE induction or applied at the first clinical signs attenuated EAE symptoms, decreased mononuclear cell infiltration in the spinal cord, and expanded the number of regulatory T cells (Tregs) (18–20). A recent clinical trial showed reduced signs of disease activity in relapsing–remitting MS patients treated with a transdermal application of a mixture of myelin peptides containing MBP85-99, MOG35-55, and PLP139-155 (21). Efficacy of specific skin-induced tolerance was also demonstrated in collagen-induced arthritis (22) and inflammatory bowel disease (23). The effectiveness of these strategies has been correlated with the presence of tolerogenic dendritic cells (DCs) and Tregs expansion. This tolerogenic condition could be hypothetically improved by the concomitant use of immunomodulatory agents such as corticosteroids, rapamycin, mycophenolate mofetil, prostaglandin E2, and vitamin D (24, 25).Vitamin D3 (VitD) works mainly through the nuclear VitD receptor (VDR) that is expressed in various immune cells such as monocytes, macrophages, DCs, and activated T and B lymphocytes (26, 27). The regulatory role of VitD in adaptive immune response also includes an inhibitory effect on DCs maturation and differentiation (28) and a Foxp3^+^ Tregs expansion (29). Direct immunomodulatory effects of VitD on CNS cells such as neural stem cells (30), astrocytes (31), and microglial cells (32) have been recently reported. VitD analogs with similar VitD immunomodulatory attributes but devoid of its side effects such as hypercalcemia and hyperphosphatemia have been developed (33–35). Paricalcitol (PARI) is a synthetic selective VDR activator used by patients with chronic renal failure to control secondary hyperparathyroidism (36). PARI retains significant immunomodulatory activity *in vitro*, characterized by impaired differentiation of immature DCs from human monocytes and a significantly decreased capacity to induce antigen-specific T cell proliferation (34). Moreover, intraperitoneal (i.p.) administration of PARI also ameliorated local inflammation and fibrosis in C56BL/6 mice through activation of Tregs and lower IL-17 levels in peritoneal cavity (35).

We recently demonstrated that i.p. MOG injection in the presence of active vitamin D was very effective as an early treatment for EAE (37). Pursuing a clinical translation of this finding and also to test PARI tolerogenicity, we evaluated the therapeutic potential of MOG plus PARI administered by epicutaneous route.

## Materials and Methods

### Experimental Design

To compare the efficacy of MOG + VitD or MOG + PARI delivered by different routes, mice were allocated into the following groups: group 1: EAE control group; groups 2 and 3: EAE mice treated with MOG + PARI or MOG + VitD by i.p. route (eight PARI or VitD doses in alternate days and two MOG doses on days 3 and 11 after EAE induction); groups 4 and 5: EAE mice treated with MOG + PARI or MOG + VitD by epicutaneous route (groups treated twice with an epicutaneous patch containing MOG + PARI or MOG + VitD on days 3 and 11); groups 6 and 7: EAE mice treated with PARI or VitD by i.p. route (eight doses in alternate days) and MOG by epicutaneous route (two doses on days 3 and 11). Clinical EAE parameters and serum calcium levels were measured during disease acute phase to compare the efficacy and a possible hypercalcemia of epicutaneous MOG associated with VitD or PARI. This first experimental design is depicted below by a timeline scheme.


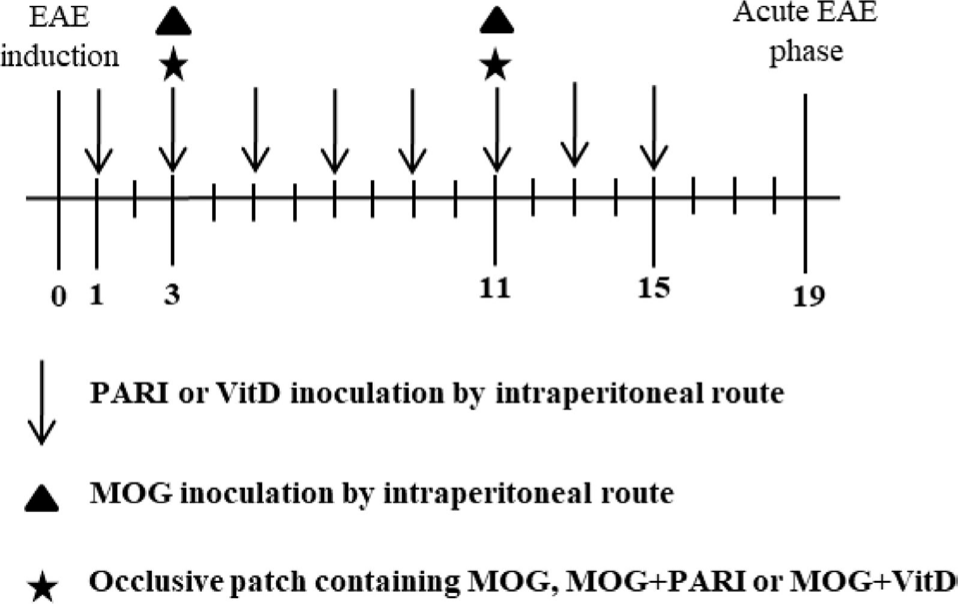


To investigate the efficacy of MOG + PARI to control EAE, mice were allocated into five experimental groups: normal mice, non-treated EAE group, and three treated EAE groups: MOG/EAE, PARI/EAE, and MOG + PARI/EAE. Three and 11 days after EAE induction, the animals were treated by epicutaneous application of MOG, PARI, or MOG + PARI in their backs. Clinical parameters were followed for 30 days. Immunological and histopathological evaluations were performed during the acute disease phase, i.e., 19 days after EAE induction. A sham control group, i.e., an EAE group that was treated with a patch containing only the MOG + PARI diluting agent, was also included in the last experiments. The adopted experimental design is illustrated below by a timeline scheme.


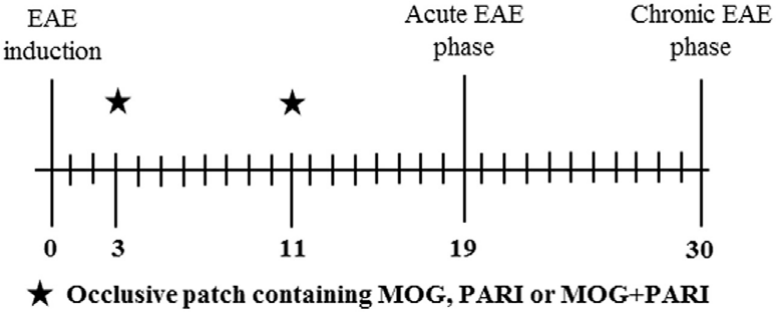


### Animals

Six-week-old female C57BL/6 mice were purchased from the University of São Paulo (USP – Ribeirão Preto, SP, Brazil) and maintained in specific pathogen-free facilities. Mice were allocated in cages (maximum six animals per cage) with sterilized food and water *ad libitum*.

### EAE Induction

Eight-week-old animals were subcutaneously injected, into the lower back, with 100 μg MOG_35–55_ peptide (MEVGWYRSPFSRVVHLYRNGK) (Genemed Synthesis Inc., San Antonio, TX, USA) emulsified with Complete Freund’s Adjuvant (Sigma Aldrich, St. Louis, MO, USA) containing 4 mg/mL of *Mycobacterium tuberculosis* (Difco, Detroit, MI, USA). Mice also received two i.p. doses, 0 and 48 h after MOG immunization, of 200 ng *Bordetella pertussis* toxin (Sigma Aldrich). Clinical score and body weight were assessed daily during 19 or 30 days according to the following criteria: 0, no symptoms; 1, limp tail; 2, hind legs weakness; 3, partially paralyzed hind legs; 4, complete hind legs paralysis; and 5, complete paralysis. The body weight variation was calculated considering the difference between the weight at the moment of EAE induction (day 0) and the one at the acute disease phase (day 19).

### Therapeutic Procedures

Mice were treated by i.p. route with 0.1 µg of 1α,25-dihydroxyvitamin D3 from Sigma Aldrich or 0.1 µg of PARI (Zemplar^®^, Abbott Laboratories, Milan, Italy), every other day during 15 days (on days 1, 3, 5, 7, 9, 11, 13, and 15) beginning 24 h after EAE induction. MOG (150 µg) was co-administered by i.p. or epicutaneous route on days 3 and 11. Some experimental protocols required concomitant application of MOG and PARI by epicutaneous route on the 3rd and 11th days after EAE induction. To perform epicutaneous immunization, mice were anesthetized with ketamine/xylazine and then shaved on their backs with a depil machine and a hair removal cream (Veet^®^, Reckitt Benckiser Healthcare, Hull, North Humberside, UK). Test substances were applied in a gauze in the center of an occlusive patch (2 cm × 2 cm) (DuoDerm Extra Thin, ConvaTec, Princeton, NJ, USA), which was then affixed to the shaved mice back. A surrounding fringe of adhesive medical tape (Micropore™, 3M United States) was used to assure the proper position and the permanence of the patch at animal’s back for 4 days.

### CNS Mononuclear Cell Isolation

Mononuclear cells infiltrated in the CNS were obtained as previously described (38). Nineteen days after EAE induction, mice were anesthetized with ketamine/xylazine and perfused with 10 mL saline solution. Brain and spinal cord were collected and digested with 2.5 mg/mL of collagenase D (Roche Applied Science, Indianapolis, IN, USA) at 37°C for 45 min. After maceration, suspensions were washed in RPMI medium (Sigma Aldrich) and centrifuged at 450 × *g* at 4°C for 10 min. Cells were resuspended in Percoll (GE Healthcare, Uppsala, Sweden) 37% and gently laid over Percoll 70%. After centrifugation at 950 × *g* for 20 min with centrifuge breaks turned off, the ring containing mononuclear cells was collected, washed in RPMI, and centrifuged again at 450 × *g* for 10 min. Cells were then resuspended in RPMI medium, counted, and analyzed. Pools from two animals were used to get enough cell number to perform the experiments.

### Cell Culture and Cytokine Quantification

Draining lymph nodes (axillary, brachial and inguinal) were collected and adjusted to 5 × 10^6^ cells/mL. Cells plated in supplemented RPMI medium (10% of fetal calf serum and 2 mM of glutamine) were restimulated *in vitro* with MOG (20 µg/mL). Cytokine levels were evaluated 48 h later by enzyme-linked immunosorbent assay in culture supernatants using IL-2, IFN-γ, and IL-10 OptEIA Sets (Cat#555148; Cat#555138; and Cat#555252) (BD Biosciences, Franklin, San Diego, CA, USA) and IL-6, IL-17, and TGF-β Duosets (Cat#DY406; Cat#DY421; and Cat#DY1679) (R&D Systems, Minneapolis, MN, USA) according to the manufacturer’s instructions.

### Microglial Cell Culture

The BV-2 cell line (BCRJ code 0356) was used to determine the cytokine production and the activation phenotype of microglial cells *in vitro*. This cell line was generated from a C57BL/6 mice brain tissue transformed by a retrovirus and retains most of the morphological, phenotypical, and functional properties of freshly isolated microglial cells (39). These cells were propagated and tested according to the study by Dulla et al. (40). Briefly, BV-2 cells were cultivated into 24-well plates at 4.0 × 10^5^ cells/well in supplemented DMEM High Glucose medium (10% of fetal calf serum and 1% of sodium pyruvate). After 2.5 h, cells were treated with PARI (1 µM) and MOG (50 µg/mL). 30 min after initial treatment, LPS (100 ng/mL) was added to cultures. Cells were incubated for 24 h in 5% CO_2_ atmosphere at 37°C. The supernatants were collected, and the levels of IFN-γ, IL-6, IL-10, and nitric oxide (NO) were determined.

### Quantification of NO Levels

Nitric oxide levels in BV-2 supernatants were measured by quantification of nitrite levels (a stable end product of NO) according to the method described by Green et al. (41). Briefly, 0.1 mL of cell-free supernatant was incubated with an equal volume of Griess reagent containing 1% sulfanilamide (Synth, Diadema, SP, Brazil), 0.1% naphthalene diamine dihydrochloride (Sigma Aldrich), and 2.5% H_3_PO_4_, at room temperature for 10 min. Nitrite accumulation was then quantified using a chemiluminescence microreader (ELx 800; BioTek Instruments Inc., Winooski, VE, USA). The concentration of nitrite was determined using sodium nitrite (Sigma Aldrich) diluted in distilled water as a standard.

### Serum Cytokine Quantification

Blood samples were collected after euthanasia by cardiac puncture and serum was obtained after centrifugation at 3,800 × *g* at room temperature for 15 min. Briefly, 25 µL serum samples were incubated with beads conjugated with fluorochromes from Cytometric Bead Array mouse Th1/Th2/Th17 cytokine kit (BD Biosciences). The acquisition of the beads was performed using a FACS Canto II flow cytometer (BD Biosciences) from Institute of Biosciences (UNESP, Botucatu, SP, Brazil) with FACS Diva software, and the data were analyzed with FCAP Array 3.0 (Soft Flow Inc., St. Louis Park, MN, USA).

### Tregs, DCs, and Microgial Cells Evaluation

Lymph nodes (axillary, brachial and inguinal) and CNS-isolated cells were collected, and the red blood cells were lysed with buffer containing NH_4_Cl. To analyze Tregs, samples were incubated with 0.2 µg PerCP-Cy5.5-labeled anti-mouse CD3 (145-2C11), 0.25 µg FITC-labeled anti-mouse CD4 (GK1.5), and 0.25 µg APC-labeled anti-mouse CD25 (PC61.5) for 30 min at 4°C. Intracellular Foxp3 transcription factor was detected using Foxp3 Staining Set (RRID: AB_465935) (eBiosciences, San Diego, CA, USA) according to manufacturer’s instructions. For myeloid DCs analysis, lymph node cells were incubated with 0.1 µg PerCP-labeled anti-mouse F4/80 (BM8), 0.25 µg FITC-labeled anti-mouse CD11c (N418), 0.03 µg APC-labeled anti-mouse MHCII (MS/114.15.2), and 0.125 µg PE-labeled anti-mouse CD86 (GL1) for 30 min at 4°C. For microglial cells, mononuclear cells isolated from CNS were incubated with 0.5 µg FITC-labeled anti-mouse CD45 (30-F11), 0.2 µg PerCP-Cy5.5-labeled anti-mouse CD11b (M1/70), 0.03 µg APC-labeled anti-mouse MHCII (MS/114.15.2), 0.15 µg PE-labeled anti-mouse CD40 (1C10), and 0.1 µg PE-Cy7-labeled anti-mouse PD-L1 (MIH5) for 30 min at 4°C. After staining, the cells were washed, resuspended in flow cytometry buffer, and fixed in 1% paraformaldehyde. Flow cytometry was performed using a FACS Canto II (BD) from Institute of Biosciences (UNESP, Botucatu, SP, Brazil), and the data were analyzed with FlowJo software (TreeStar, Ashland, OR, USA).

### PCR Assays

RNA was extracted from CNS-isolated cells with TRIzol™ reagent (Life Technologies, Austin, TX, USA). 200 ng of RNA was converted to cDNA using High Capacity cDNA Reverse Transcription kit (Life Technologies) according to the manufacturer’s instructions. Expression of Foxp3 (Mm00475162_m1) and RORc (Mm01261022_m1) was analyzed in comparison to GAPDH (Mm99999915_g1, housekeeping gene) levels. Real time-PCR reactions were performed using TaqMan™ Gene Expression Assays according to manufacturer’s recommendations (Applied Biosystems, Carlsbad, CA, USA). Levels of gene expression were represented as relative fold difference by using the method of delta threshold (2^−ΔΔCt^).

### Inflammatory Process in the CNS

The histopathological analysis was performed 19 days after EAE induction, during the acute phase of the disease. After euthanasia, lumbar spinal cord samples were removed and fixed in 10% neutral buffered formalin. Tissues were dehydrated in graded ethanol and embedded in Paraplast Plus (McCormick, St. Louis, MO, USA). Serial sections with 5-µm thickness were cut and stained with hematoxylin and eosin. Five photomicrographs were obtained from each animal with a Nikon microscope. A semiquantitative analysis of CNS inflammation was performed according to the following criteria, as previously described (42): 0, no infiltrates; 1, partial meningeal infiltration; 2, pronounced meningeal infiltration, and 3, pronounced meningeal and some parenchymal infiltration.

### Myelin Sheath Analysis

Myelin sheath thickness was assessed in lumbar spinal cord samples during the EAE acute phase by transmission electron microscopy. Mice were anesthetized with ketamine/xylazine and perfused intracardially with 10 mL Karnovsky solution. After removing spinal cords from the vertebral canal, tissues were fixed during 24 h in Karnovsky solution at 4°C. Specimens were rinsed in phosphate buffer and treated with 1% osmium tetroxide for 2 h. After another rinse in phosphate buffer, tissues were treated for 2 h with 0.5% uranyl acetate followed by dehydration in graded acetone series and finally embedded in Araldite resin. To assure a representative comparison in the myelin sheath among the experimental groups, a corresponding area of lumbar spinal cord was used to obtain 1-µm thick section. From this area located in the more infiltrated region, ultrathin sections with 90 nm were mounted on 150 mesh formvar-coated copper grids (Electron Microscopy Sciences, Hatfield, PA, USA) and contrasted with 1% uranyl acetate for 20 min and lead citrate solution for 10 min. Sections were examined on a Tecnai Spirit Transmission Electron Microscope (FEI Company, OR, USA) at the Electron Microscopy Center of the Institute of Biosciences (UNESP, Botucatu, SP, Brazil). Ten images per mouse were taken at 2,900× magnification by using a digital camera connected to electron microscope.

### Blood–Spinal Cord Barrier Permeability Assay

To perform the spinal cord barrier permeability assay, we used the Naflu test based on the methodology described by Christy et al. (43). Twelve days after EAE induction, mice were i.p. inoculated with 100 µL fluorescein sodium (NaFlu, Sigma Aldrich). After 20 min, the animals were anesthetized with ketamine/xylazine, and blood samples were collected by cardiac puncture with heparinized syringe. After that, mice were perfused with 10 mL saline solution, and the whole spinal cord sections were collected in microtubes and homogenized with 400 µL 0.9% saline solution. After centrifugation at 9,000 rpm for 10 min at 22°C, the supernatant of spinal cord and the plasma were added in black 96-well immuno plates. The fluorescence was measured in BioTek Synergy™ 4 hybrid microplate reader (emission 485 nm/excitement 528 nm). Results were expressed in relative fluorescence units (RFUs). The following equation was used to evaluate NaFlu incorporation in the spinal cord of each animal: NaFlu incorporation = (spinal cord RFU/spinal cord weight)/(plasma RFU/blood volume).

### Statistical Analysis

Data were expressed as mean ± SD or with median and interquartile (25–75%) ranges. To test normality of data, results were analyzed by Shapiro–Wilk’s test. Comparisons between experimental groups were made by one-way ANOVA followed by Tukey’s test for parametric variables and by Kruskal–Wallis test followed by Dunn’s test for non-parametric variables. Chi-square and Fisher exact test were performed for EAE incidence. Statistical analysis was accomplished with SigmaPlot Software Version 12.0 (Systat Software Inc., San Jose, CA, USA), and *p* < 0.05 was considered significant.

## Results

### Administration of MOG Associated With VitD or Analog by Distinct Routes Controlled EAE Development

**Figure 1:**
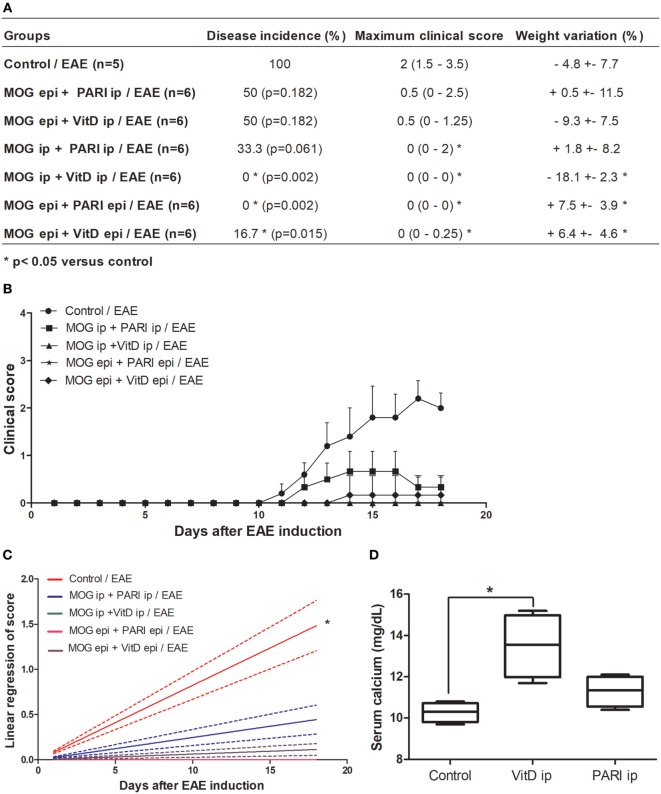
Effect of MOG + VitD or MOG + PARI on experimental autoimmune encephalomyelitis (EAE) development. C57BL/6 mice were submitted to EAE induction and treated with MOG + VitD or MOG + PARI by different routes. Disease incidence, maximum clinical score and body weight variation **(A)**, kinetics of clinical scores **(B)**, and linear regression analysis of clinical score **(C)** were daily recorded until the EAE acute phase. Serum calcium levels were determined in mice treated with vitamin D3 (VitD) or paricalcitol (PARI) by i.p. route 1 day after the end of treatment **(D)**. The results are expressed as mean ± SD or medians (25–75% ranges) of 5–6 mice per group. *Difference between EAE and treated groups. *p < 0.05.

We recently described that MOG + VitD association inhibits EAE development in both prophylactic (44) and therapeutic (37) approaches. In both investigations, MOG and VitD were administered by i.p. route. Considering that VitD and PARI share immunomodulatory activities *in vitro* (34), that PARI does not trigger hypercalcemia, and that i.p. route is not suitable for human use, it was necessary to evaluate the efficacy of other routes and also the suitability of PARI as a VitD substitute. EAE mice were treated every other day during 15 days with VitD or PARI, and MOG was co-administered on days 3 and 11. This combination of MOG with VitD or PARI was then tested by three different protocols whose objective was to compare the efficacy of different routes. As depicted in Figure 1A, the administration of MOG and VitD or PARI by the same route, i.e., intraperitoneal or epicutaneous, was more efficient to control EAE development than their application by distinct routes. When epicutaneous MOG was associated with VitD or PARI by i.p. route, the disease incidence was still very elevated (around 50%). These data also indicated that PARI does not trigger body weight loss (Figure 1A) neither hypercalcemia (Figure 1D) as occurs with VitD. Comparison of clinical scores, showed in Figure 1B, reveals that MOG + VitD or MOG + PARI delivered by intraperitoneal or epicutaneous route control the disease in a very similar way with no statistical differences (Figure 1C).

### Therapeutic Efficacy of Epicutaneous MOG Was Increased by PARI

**Figure 2:**
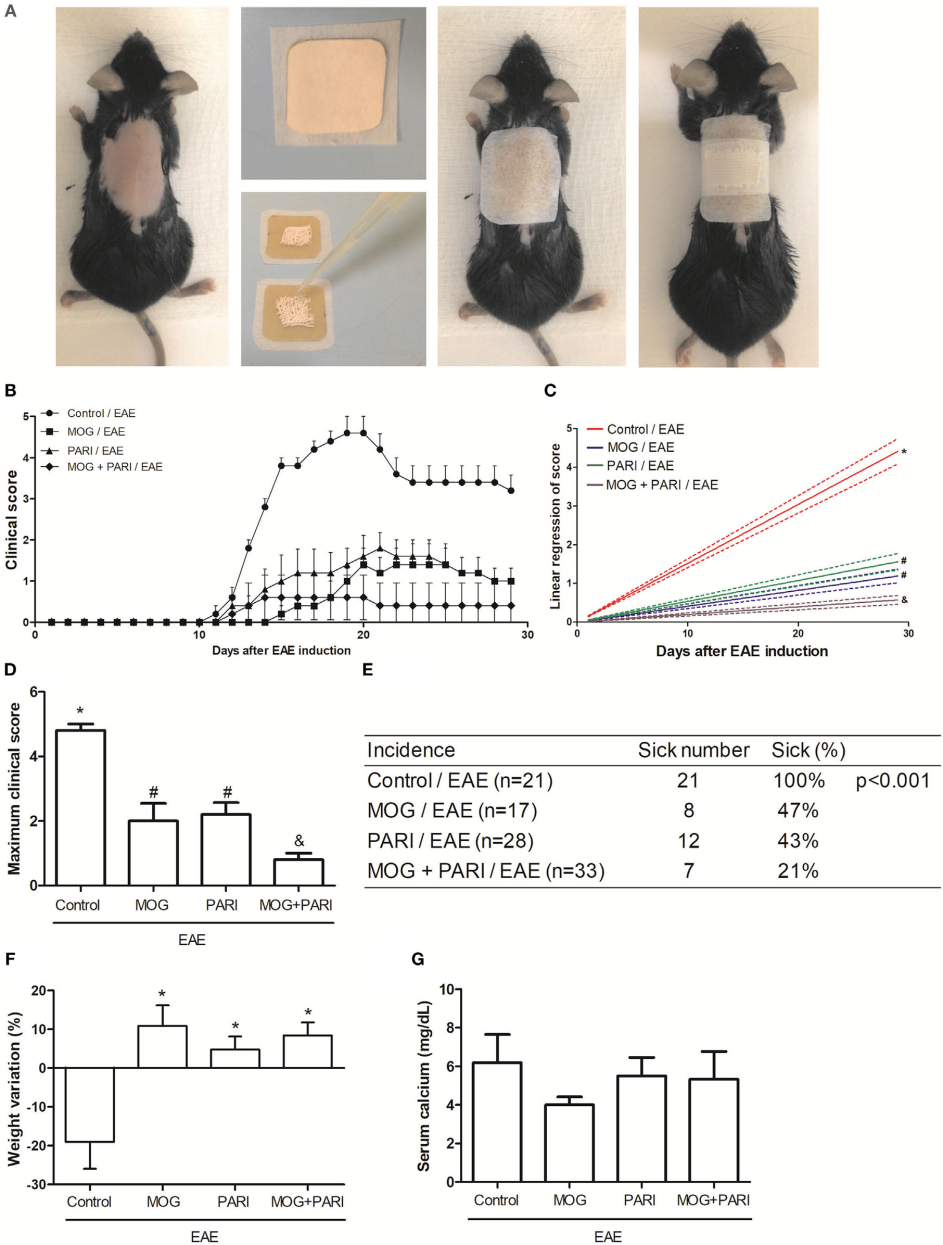
Efficacy of epicutaneous MOG + PARI association on experimental autoimmune encephalomyelitis (EAE) development. C57BL/6 mice were submitted to EAE induction and treated with a MOG + PARI association by epicutaneous route at days 3 and 11 after disease induction. Scheme of the occlusive patch applied in mice shaved back **(A)**, kinetics of clinical scores **(B)**, linear regression analysis of clinical score **(C)**, maximum clinical scores **(D)**, incidence **(E)**, and body weight variation **(F)** were daily recorded until the EAE recovery phase. The percentage of weight variation was determined during EAE acute phase, i.e., from day 0 to 19. Serum calcium levels were determined 1 day after the end of treatment **(G)**. The results are expressed as mean ± SD (6 mice/group), and one experiment representative of four is shown. Different symbols indicate significant difference between groups **(C,D)**. *Difference between EAE and treated groups **(D)**. *p < 0.05.

As i.p. MOG + PARI was as efficient as epi MOG + PARI and considering that epicutaneous route could be easily translated to human application, this procedure was chosen to be investigated in more details. Initially, to certify that administration of PARI would improve MOG efficacy when the two substances were administered concomitantly by the same epicutaneous route, mice were submitted to EAE induction, and after 3 and 11 days, they received an occlusive patch containing MOG, PARI, or MOG plus PARI (Figure 2A). Animals were daily monitored for disease development during 30 days until EAE chronic phase. Co-administration of MOG + PARI, both by epicutaneous route, determined a much milder disease in comparison with MOG or PARI administration alone (Figure 2B). The protective effect of co-administration was supported by significant lower clinical scores (Figures 2C,D), no body weight loss (Figure 2F) and a clear decreased disease incidence (Figure 2E). As predicted, serum calcium levels in PARI and MOG + PARI groups were not altered (Figure 2G). Sham treatment did not protect the animals (data not shown).

### Epicutaneous MOG + PARI Association Modulated Peripheral Immune Response

**Figure 3:**
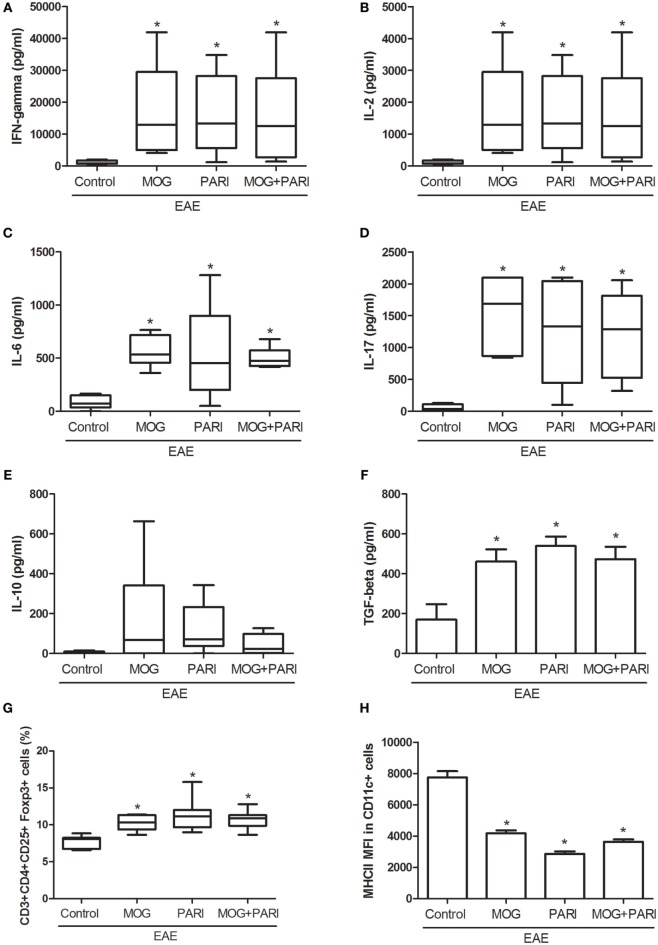
Effect of MOG + PARI association on peripheral immune response. C57BL/6 mice were submitted to experimental autoimmune encephalomyelitis (EAE) induction and treated with a MOG + PARI association by epicutaneous route at days 3 and 11 after disease induction. Peripheral immunological parameters were assessed at acute EAE phase (19th day). IFN-γ **(A)**, IL-2 **(B)**, IL-6 **(C)**, IL-17 **(D)**, IL-10 **(E)**, and TGF-β **(F)** levels were measured in regional lymph node cell cultures (5 × 10^6^ cells/mL) stimulated with MOG (20 µg/mL). The percentage of Foxp3^+^ Tregs in 100,000 acquired events was determined in regional lymph nodes **(G)**. Mean fluorescence intensity (MFI) of MHCII in F4/80-CD11c^+^ DCs in 500,000 acquired events was also measured in draining lymph nodes **(H)**. The results are expressed as mean ± SD or medians (25–75% ranges) of six mice per group, and one experiment representative of two is shown. *Difference between EAE and treated groups, *p < 0.05.

**Figure 4:**
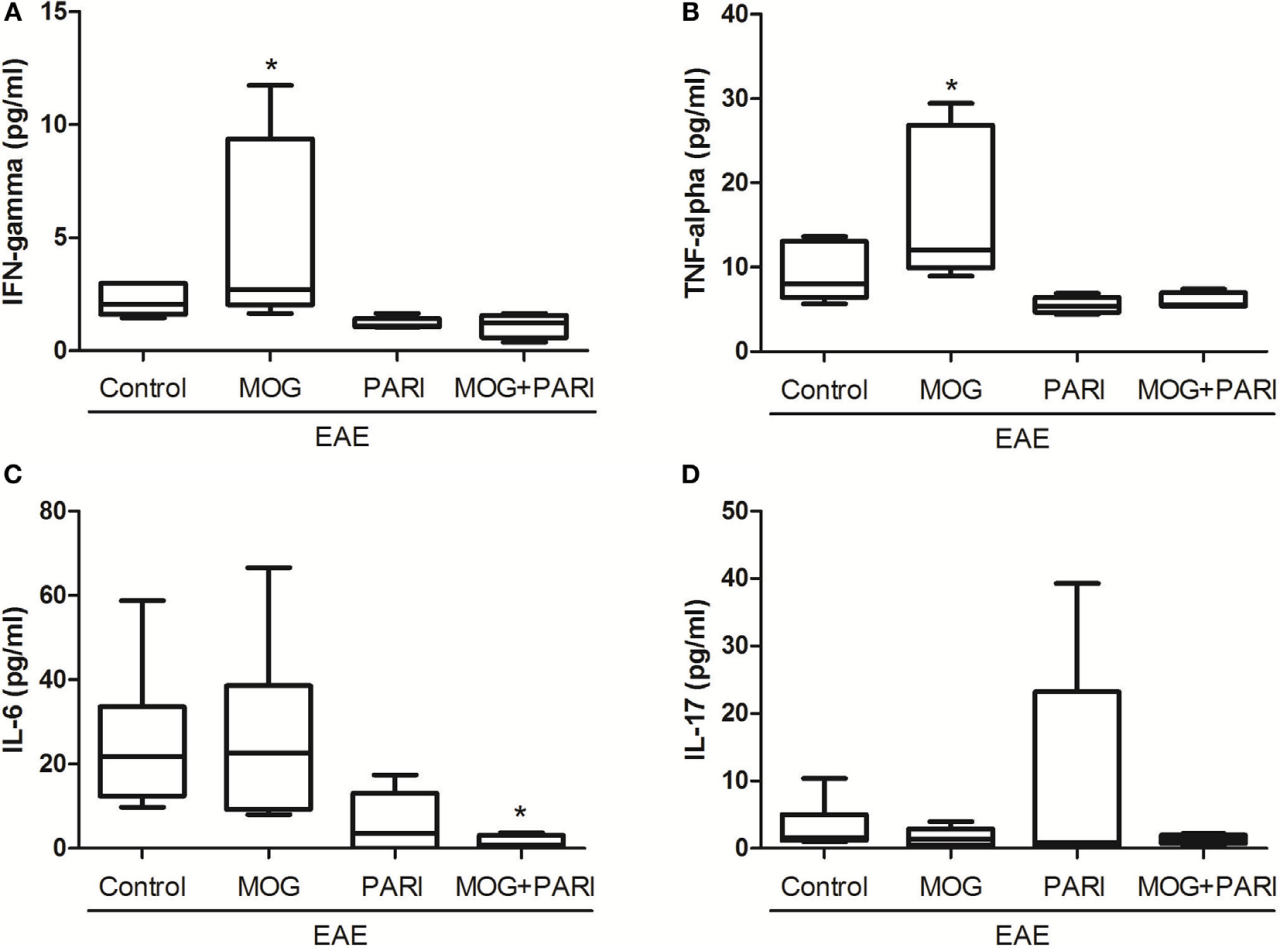
Effect of MOG + PARI association on serum cytokine levels. C57BL/6 mice were submitted to experimental autoimmune encephalomyelitis (EAE) induction and treated with a MOG + PARI association by epicutaneous route at days 3 and 11 after disease induction. Serum cytokines were assessed at acute EAE phase (19th day). IFN-γ **(A)**, TNF-α **(B)**, IL-6 **(C)**, and IL-17 **(D)** levels were measured in serum samples by flow cytometry. The results are expressed as medians (25–75% ranges) of six mice per group, and one experiment representative of two is shown. *Difference between EAE and treated groups, *p < 0.05.

As a recent work from our group indicated downregulation of encephalitogenic cytokine production in the spleen of EAE mice submitted to therapy with VitD + MOG association *via* i.p. route (37), we expected that this alternative therapeutic route would have the same effect. Unexpectedly, epicutaneous therapy with MOG, PARI, or MOG + PARI similarly increased pro-inflammatory cytokine production by lymph node cell cultures re-stimulated *in vitro* with MOG, when compared to the EAE control group (Figures 3A–D). Nonetheless, a regulatory immune profile was equally present in the lymph nodes from treated EAE mice, characterized by higher levels of TGF-β (Figure 3F), elevated percentages of Foxp3^+^ Tregs (Figure 3G), and lower MHCII fluorescence intensity in DCs (Figure 3H). Higher amounts of IL-10 were produced by the three treated EAE groups (Figure 3E). The levels of serum cytokines did not follow the same pattern found in lymph node cell cultures. MOG increased IFN-γ (Figure 4A) and TNF-α (Figure 4B), but did not alter IL-6 (Figure 4C) and IL-17 (Figure 4D). Nonetheless, PARI treatment slightly decreased IFN-γ (Figure 4A), TNF-α (Figure 4B), and IL-6 (Figure 4C) serum levels. When PARI was associated with MOG, the reduction of encephalitogenic cytokines in the serum was more pronounced, being the reduction in IL-6 production statistically significant (Figure 4C).

### Epicutaneous MOG + PARI Association Reduced Inflammation and Demyelination in the CNS

**Figure 5:**
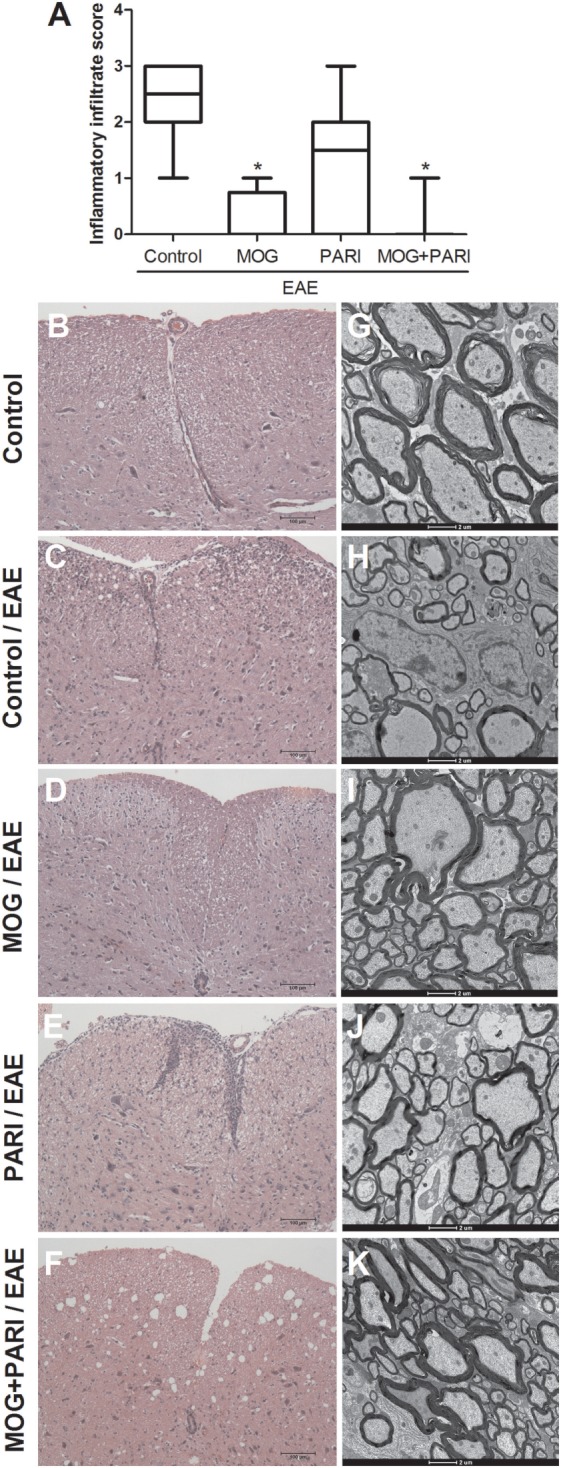
Control of inflammation and demyelination in the central nervous system by MOG + PARI association. C57BL/6 mice were submitted to experimental autoimmune encephalomyelitis (EAE) induction and treated with a MOG + PARI association by epicutaneous route at days 3 and 11 after disease induction. A semiquantitative analysis **(A)** was used to assess the inflammatory infiltration in the lumbar spinal cord at acute EAE phase (19th day). Representative sections stained with hematoxylin and eosin from all groups **(B–F)**. Corresponding areas of lumbar spinal cord were used to assess myelin sheath swollen using transmission electron microscopy **(G–K)**. The results are expressed as medians (25–75% ranges) of six mice per group. *Difference between EAE and treated groups, *p < 0.05.

Bearing in mind that EAE is characterized by infiltration of immune cells in the CNS and predominant demyelinated axons in the acute phase of the disease (45), the therapeutic effect of MOG + PARI association could be related to a milder CNS pathology. Stained lumbar spinal cord sections were semiquantitatively evaluated concerning the degree of inflammation during the EAE acute phase (Figure 5A). Although the EAE group presented a pronounced inflammatory process in the meninges and in the spinal cord parenchyma (Figure 5C), MOG and MOG + PARI treatments significantly reduced the extension of the inflammatory infiltrate as illustrated at Figures 5D,F. Nonetheless, the degree of inflammation in the lumbar spinal cord of mice treated with PARI was similar to the one found in non-treated EAE animals (Figure 5E). By using electron microscopy, we found that MOG (Figure 5I) and MOG + PARI (Figure 5K) association conserved nerve fibers with myelin sheath, whereas PARI treatment (Figure 5J) showed areas with demyelinated axons (complete loss of the myelin sheath) similar to EAE control group (Figure 5H). Spinal cord sections from normal mice (control) stained with hematoxylin and eosin or prepared for electron microscopy are represented in Figures 5B,G respectively.

### Epicutaneous MOG + PARI Association Upregulated Tregs Proportion in the CNS

**Figure 6:**
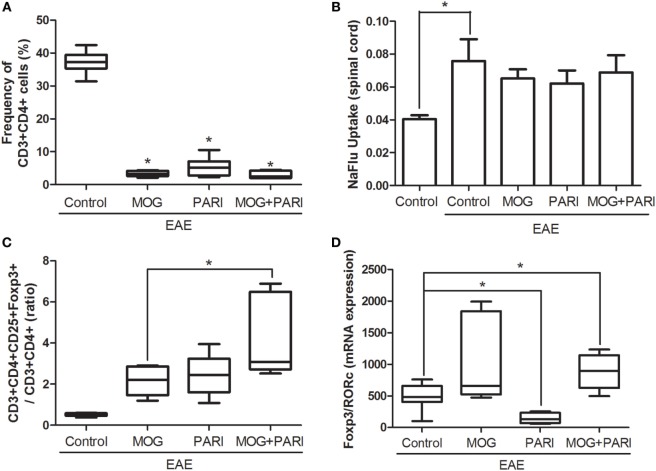
MOG + PARI association upregulates regulatory T cells in the central nervous system (CNS). C57BL/6 mice were submitted to experimental autoimmune encephalomyelitis (EAE) induction and treated with a MOG + PARI association by epicutaneous route at days 3 and 11 after disease induction. The phenotype of T cells was determined in the CNS at acute EAE phase (19th day), and a blood–spinal cord barrier permeability test was done at the preclinical stage (12th day). Percentage of CD3^+^CD4^+^ cells in 100,000 acquired events **(A)**, NaFlu uptake in spinal cord **(B)**, ratio between percentage of Foxp3 cells and CD4^+^ cells **(C)**, and ratio between mRNA expression for Foxp3 and RORc **(D)**. The results are expressed as mean ± SD or medians (25–75% ranges) of six mice per group, and one experiment representative of two is shown. *Difference between EAE and treated groups **(A,D)**, *difference between control and EAE group **(B)**, *difference among treated groups **(C)**, *p < 0.05.

Reduced inflammation revealed by histopathological analysis was further confirmed by determination of the CD3^+^CD4^+^ percentage, by cytometric analysis, in cells eluted from the CNS (Figure 6A). A NaFlu test made during the preclinical phase indicated that reduced inflammation was not due to a decreased blood–spinal cord barrier permeability (Figure 6B). Considering that immunomodulatory properties of vitamin D and its analogs are usually correlated with the suppressive activities of Tregs (34, 46), mRNA expression for Foxp3 and RORc transcription factors and the frequency of CD3^+^CD4^+^CD25^+^Foxp3^+^ cells were evaluated in mononuclear cells recovered from the CNS during the acute phase of the disease. The more efficient therapeutic effect of MOG + PARI treatment in EAE development coincided with a higher ratio between the percentages of CD25^+^Foxp3^+^ and CD3^+^CD4^+^ cells (Figure 6C). Besides, MOG + PARI treatment determined a significantly augmented ratio between relative mRNA expression for Foxp3 and RORc (Figure 6D).

### MOG + PARI Association Modulated Microglial Cells both *In Vivo* and *In Vitro*

**Figure 7:**
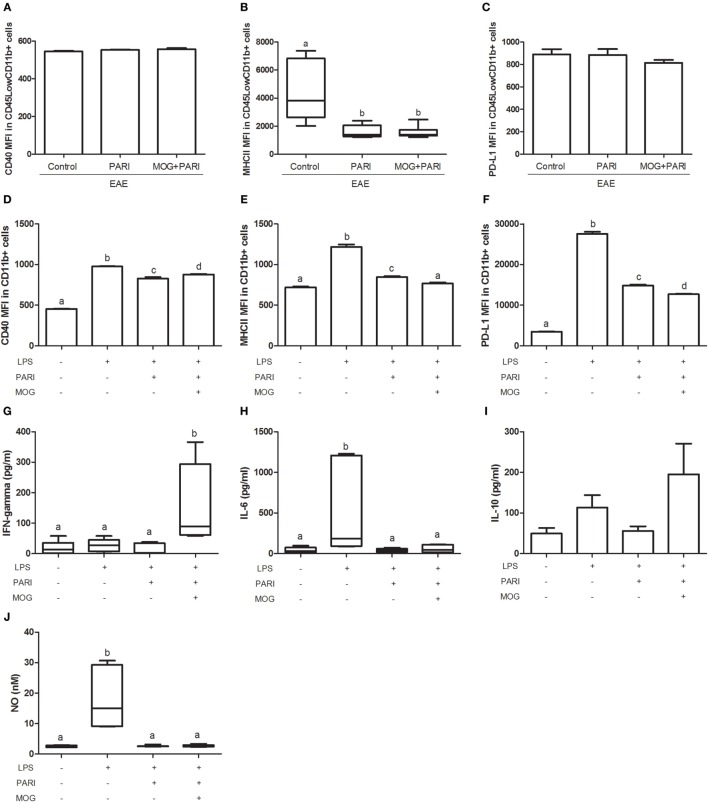
MOG + PARI association modulates microglial cells. C57BL/6 mice were submitted to experimental autoimmune encephalomyelitis (EAE) induction and treated with a MOG + PARI association by epicutaneous route at days 3 and 11 after disease induction. The activation status of microglial cells eluted from central nervous system **(A–C)** at acute EAE phase (19th day) and of BV-2 cell line *in vitro* activated with LPS after pretreatment with PARI and MOG **(D–J)**. Mean fluorescence intensity (MFI) of CD40 **(A)**, MHCII **(B)**, and PD-L1 **(C)** in CD45^Low^CD11b^+^ cells in 200,000 acquired events. MFI of CD40 **(D)**, MHCII **(E)**, and PD-L1 **(F)** in CD11b^+^ cells in 200,000 acquired events. IFN-γ **(G)**, IL-6 **(H)**, IL-10 **(I)**, and nitric oxide (NO) **(J)** levels were measured in the supernatant of microglial cell cultures. The results are expressed as mean ± SD or medians (25–75% ranges) of six mice per group for *in vivo* experiment **(A–C)** and one result of three repetitions *in vitro* assays **(D–J)**. Different letters indicate significant difference between groups, *p* < 0.05.

As VitD is able to reach the CNS and to interact with microglial cells (32), a similar characteristic was expected from the analog. This possibility was investigated in microglial cells eluted from treated EAE mice and also in BV-2 cell line. As demonstrated in Figure 7B, microglial cells eluted from the CNS of mice treated with PARI or MOG + PARI showed a similar and significant reduction in the mean fluorescence intensity (MFI) of MHCII. CD40 (Figure 7A) and PD-L1 (Figure 7C) MFI expression were not affected. This downmodulatory effect was confirmed by showing that PARI and MOG + PARI addition to BV-2 cells previously stimulated with LPS significantly decreased MFI MHCII expression (Figure 7E). In this experimental *in vitro* procedure, a significant reduction in CD40 (Figure 7D) and PD-L1 (Figure 7F) were also observed. The *in vitro* activity of MOG + PARI revealed other possible regulatory pathways involving cytokines and NO. In this case, MOG + PARI association decreased IL-6 (Figure 7H) and NO (Figure 7J) and increased IFN-γ (Figure 7G) and IL-10 (Figure 7I).

## Discussion

The proof of concept that MOG + VitD association is highly tolerogenic in mice with EAE was already described by our group. In this previous investigation, we demonstrated that the association of MOG with active VitD, delivered by i.p. route, controlled EAE development in C57BL/6 mice (37). Nonetheless, this efficient therapeutic approach was achieved by using an artificial immunization route and collateral effects as hypercalcemia and weight loss were identified after VitD inoculation. The potential translation of this finding to human patients would demand, however, a more accepted route of administration. A VitD analog devoid of side effects would also be required. In this context, the therapeutic effect of MOG plus VitD and MOG plus PARI association delivered by i.p. or epicutaneous routes were compared. Considering that the rational of these combinations was focused on the possible immunomodulatory effect of VitD/PARI on APCs, we also asked if the two substances needed to be administered by the same route. MOG + VitD and MOG + PARI by i.p., epicutaneous or a combination of these two routes (MOG epi + VitD i.p.), were all able to reduce EAE incidence, maximum clinical scores, and body weight loss. However, observing mainly disease incidence and maximum clinical scores that are relevant parameters, the association of MOG epi with VitD or PARI i.p. was clearly less effective, showing that the higher efficiency of this strategy indeed requires administration of both substances by the same route.

Considering that epicutaneous application of MOG + PARI was as efficient as MOG + VitD, that PARI was devoid of the tested deleterious effects, that Zemplar^®^ is a drug approved by FDA for human use, and that the epicutaneous route would be easily accepted for human use, this schedule was adopted for a more detailed investigation. The efficacy of MOG, PARI, or MOG + PARI delivered by a patch applied to the shaved animal’s back was then compared. Even though MOG and PARI alone were effective, their association was clearly better. Lower clinical scores analyzed by both linear regression and maximum clinical score, and also lower disease incidence supports the higher efficacy of MOG + PARI. The therapeutical use of CNS peptides is not a novelty. Skin-induced tolerance with myelin peptides was already described in mice and human trials (18, 21). This treatment was well tolerated by patients with relapsing–remitting MS and was also able to reduce disease activity (21). In this sense, the contribution of this work is the demonstration that a VitD analog can improve this tolerogenic therapeutic strategy.

This more effective strategy was accompanied by lower levels of serum pro-inflammatory cytokines during acute EAE phase. Unexpectedly, an increased production of cytokines was found in cultures from draining lymph nodes after *in vitro* re-stimulation with MOG. This elevated cytokine production also diverged from our previous findings (37). It is important to highlight, however, that both the therapeutic route and the peripheral lymphoid organ analyzed in these two investigations were distinct. In the previous publication (37), we employed an i.p. route for therapy and tested cytokine production by the spleen, whereas in this study, we quantified cytokines in lymph node cell cultures after an epicutaneous immunization. In spite of this elevated production of pro-inflammatory cytokines, the three therapeutic procedures determined significant increases in MOG-induced TGF-β production and also in the percentage of Foxp3^+^ Tregs. Considering that this T cell subset and this mediator have been highly emphasized as master regulators of inflammation in the CNS from both, EAE and MS patients (6, 47–49), they could be involved in the protection determined by these three procedures. The possibility that this protective effect is being mediated, in some degree, by Tregs induced by both MOG and VitD analog finds support in the literature. The first report that CNS antigen delivered through the skin induces T cells with suppressive activities in EAE mice was described by Szczepanik et al. (18). These authors demonstrated that Tregs induced by epicutaneous immunization with MBP inhibit T cell antigen-specific proliferation *in vitro via* the production of TGF-β. This same group also showed that the efficacy of epicutaneous application of MBP in EAE mice was related to induction of CD4^+^CD8^+^ double-positive suppressor T cells. This ability of VitD or its analogs to induce Tregs when applied to the skin has been also demonstrated with different experimental models. By using a human skin explant system, Bakdash et al. (50) demonstrated that the intradermal application of VitD enhanced the migration of tolerogenic DCs, promoting the development of Foxp3^+^ Tregs and abolishing IFN-γ production. Exposure of the skin to a VitD analog named calcipotriol, before immunization with ovalbumin protein and CpG adjuvant, prevented antigen-specific CD8^+^ T cell proliferation through induction of Foxp3^+^ Tregs (51). Interestingly, a VitD analog designated Ro 26-2198 was able to reduce diabetes incidence in NOD mice enhancing the frequency and suppressive activity of Tregs in pancreatic lymph nodes (52), supporting therefore the likelihood that Tregs are related to MOG + PARI protective effect. In a mouse model of peritoneal fibrosis, PARI reduced peritoneal inflammation and fibrosis through the activation of CD4^+^ and CD8^+^ Tregs in the peritoneal cavity (35).

Considering that tolerogenic DCs play a key role in immune tolerance by increasing Tregs expansion, we investigated whether our therapeutic strategy resulted in an impairment of DCs maturation. Indeed, all three therapeutic approaches determined lower expression of MHCII in DCs, which could prime for a regulatory profile expansion in the regional lymph nodes. This immature state of DCs has been characterized by lower surface expression of costimulatory and MHCII molecules (28, 53). The contribution of low MHCII expression to tolerance induction was recently validated by Ray et al. (54). These authors demonstrated that lack of R-Ras, a regulator of many cellular processes, resulted in attenuated EAE development. They also observed that R-Ras absence resulted in MHCII^Low^ expression in DCs and a pronounced proliferation of natural Tregs in the periphery. As topical VitD can generate tolerogenic DCs able to enhance the suppressive function of Tregs residing in skin-draining lymph nodes (55), epicutaneous PARI administration could be involved in the immature DCs state observed in our study.

Despite the high pro-inflammatory immune response in the periphery, the migration of CD4^+^ T cells to the CNS was significantly reduced (around 75%) in animals treated with MOG, PARI, and MOG/PARI. Our data demonstrated that MOG-reactive Th cells are successfully generated in the presence of MOG/PARI treatment, secreting both pro-inflammatory and anti-inflammatory cytokines in draining lymph nodes. However, lower levels of encephalitogenic cytokines were detected in the serum, suggesting that these activated T cells could be retained in this lymphoid organ. A similar phenomenon was described for fingolimod that causes lymphopenia due to lymphocyte sequestration in the secondary lymphoid tissues (56). PARI administration could be somehow responsible for reduced cell migration to the CNS. Recent findings demonstrated that oral treatment with VitD prevented Th cell migration to the CNS in EAE mice, despite intense activation of pathogenic T cells in the periphery (57). These authors also described that VitD maintained the integrity of BBB and determined less expression of the chemokine receptor CXCR3 that is directly involved in T cell transendothelial migration to the CNS. Differently from this finding, the result of the NaFlu test suggests that PARI is not able to stabilize the blood–spinal cord barrier. Treatment with MOG/PARI association not only prevented accumulation of inflammatory infiltrates in the lumbar spinal cord but also reduced myelin sheath damage. As previously described by Soellner et al. (45), we also observed extensive inflammation associated with areas of demyelinated axons in the CNS during the acute disease phase. Treatment with MOG and MOG/PARI clearly decreased the number of demyelinated axons in the CNS.

Despite the fact that epicutaneous MOG therapy alone was capable to decrease CNS pathology, its association with PARI increased parameters usually associated with a regulatory activity in the CNS. This therapeutic strategy significantly increased the frequency of Foxp3^+^ Tregs, which is one of the most targeted cells during EAE and MS immunotherapy (47, 58, 59). The ratio of Foxp3/RORc mRNA expression was also clearly augmented in the CNS of MOG + PARI group. Numerous findings support the view that IL-17 plays an essential role in autoimmune CNS inflammation (3, 6, 60). Moreover, to avoid trafficking of Th17 cells to the CNS is a pivotal approach to control EAE development. Similar alterations were triggered by i.p. PARI administration in a mouse model of peritoneal fibrosis. This treatment reduced IL-17 production and activated a regulatory response, characterized by increased numbers of both CD4^+^ and CD8^+^ Tregs in the peritoneal cavity where the inflammatory process was taking place (35).

Having in mind that VitD reaches the CNS (32) where it plays multiple neuroprotective effects (61) by both genomic and non-genomic mechanisms (62), we reasoned that PARI could also play a direct effect on CNS. This hypothesis was checked by evaluating the *in vivo* and *in vitro* effect of PARI in microglial cells. Microglial cells eluted from mice treated with PARI or MOG + PARI exhibited a significant reduction in the MFI of MHCII expression emphasizing the possibility that PARI is reaching the CNS and contributing to downmodulate microglia activity. By using an *in vitro* system comprised by BV-2 cells treated with PARI or MOG + PARI before stimulation with LPS, we confirmed this reduction in MHCII expression and also on the ability to produce NO, which are two parameters indicative of microglial activation in inflammatory diseases of the CNS (40, 63–66). This *in vitro* model also suggested that other molecules as PD-L1 and IFN-γ could contribute to this presumed direct PARI effect. The fact that PARI lowered the PD-L1 expression levels was initially unexpected because the majority of the published data shows that microglial cells suppress T cells responses through higher PD-L1 expression (67, 68). A few papers suggest, however, that this inhibitory effect could be associated with soluble PD-L1, and therefore, a decreased expression of these molecules would be observed (69). The significantly elevated levels of IFN-γ produced by microglial cells stimulated with MOG and LPS in the presence of PARI was, in the light of classical knowledge, unexpected. IFN-γ has been usually understood as a pro-inflammatory damage mediator of both, EAE and MS (70, 71). Nevertheless, more precise and refined procedures lately revealed that pro-inflammatory cytokines, including IFN-γ, can display these seemingly paradoxical immunoregulatory attributes (72).

In summary, our data demonstrate that epicutaneous application of MOG plus a VitD non-calcemic analog was highly effective to control EAE development. The possible transference of this knowledge to MS patients still needs further preclinical studies, but this possibility is highly strengthened by the fact that both CNS antigens and VitD are already being individually employed in clinical trials.

## Ethics Statement

The animals were manipulated in accordance with the brazilian legislation that is regulated by the National Council for the Control of Animal Experimentation (CONCEA) and by the Ethical Principles in Animal Research formulated by the Brazilian Society of Science in Laboratory Animals. The whole experimental protocol was also approved by the Institute of Biosciences Ethics Committee on Use of Animals (CEUA – protocol number 498), UNESP, Botucatu, SP, Brazil.

## Author Contributions

SZ-P and AS conceived and designed the experiments. SZ-P, LM, TF-S, LI, and TF performed the experiments. SZ-P, LM, TF-S, LI, and TF contributed to analysis tools. SZ-P, LM, TF-S, LI, TF, and AS analyzed the data. SZ-P, LM, TF-S, LI, TF, and AS approved the final version to be published. SZ-P and AS wrote the paper.

## Conflict of Interest Statement

We declare that the research was conducted in the absence of any commercial or financial relationships that could be construed as a potential conflict of interest.

## References

[1] Lassmann H Bruck W Lucchinetti CF. The immunopathology of multiple sclerosis: an overview. Brain Pathol (2007) 17(2):210–8. 10.1111/j.1750-3639.2007.00064.x 17388952 PMC8095582

[2] Hemmer B Cepok S Nessler S Sommer N. Pathogenesis of multiple sclerosis: an update on immunology. Curr Opin Neurol (2002) 15(3):227–31. 10.1097/00019052-200206000-00001 12045717

[3] Rostami A Ciric B. Role of Th17 cells in the pathogenesis of CNS inflammatory demyelination. J Neurol Sci (2013) 333(1–2):76–87. 10.1016/j.jns.2013.03.002 23578791 PMC3726569

[4] O’Connor RA Prendergast CT Sabatos CA Lau CW Leech MD Wraith DC Cutting edge: Th1 cells facilitate the entry of Th17 cells to the central nervous system during experimental autoimmune encephalomyelitis. J Immunol (2008) 181(6):3750–4. 10.4049/jimmunol.181.6.3750 18768826 PMC2619513

[5] Bradl M Lassmann H Oligodendrocytes: biology and pathology. Acta Neuropathol (2010) 119(1):37–53. 10.1007/s00401-009-0601-5 19847447 PMC2799635

[6] Fletcher JM Lalor SJ Sweeney CM Tubridy N Mills KH T cells in multiple sclerosis and experimental autoimmune encephalomyelitis. Clin Exp Immunol (2010) 162(1):1–11. 10.1111/j.1365-2249.2010.04143.x 20682002 PMC2990924

[7] Brück W The pathology of multiple sclerosis is the result of focal inflammatory demyelination with axonal damage. J Neurol (2005) 252(5):v3–9. 10.1007/s00415-005-5002-7 16254699

[8] Hemmer B Kerschensteiner M Korn T. Role of the innate and adaptive immune responses in the course of multiple sclerosis. Lancet Neurol (2015) 14(4):406–19. 10.1016/S1474-4422(14)70305-9 25792099

[9] Kawachi I Lassmann H Neurodegeneration in multiple sclerosis and neuromyelitis optica. J Neurol Neurosurg Psychiatry (2017) 88(2):137–45. 10.1136/jnnp-2016-313300 27671902

[10] Castro-Borrero W Graves D Frohman TC Flores AB Hardeman P Logan D Current and emerging therapies in multiple sclerosis: a systematic review. Ther Adv Neurol Disord (2012) 5(4):205–20. 10.1177/1756285612450936 22783370 PMC3388530

[11] Miron VE Schubart A Antel JP. Central nervous system-directed effects of FTY720 (fingolimod). J Neurol Sci (2008) 274(1–2):13–7. 10.1016/j.jns.2008.06.031 18678377

[12] Miller A Lider O Roberts AB Sporn MB Weiner HL. Suppressor T cells generated by oral tolerization to myelin basic protein suppress both in vitro and in vivo immune responses by the release of transforming growth factor beta after antigen-specific triggering. Proc Natl Acad Sci U S A (1992) 89(1):421–5. 10.1073/pnas.89.1.421 1370356 PMC48249

[13] Hilliard B Ventura ES Rostami A. Effect of timing of intravenous administration of myelin basic protein on the induction of tolerance in experimental allergic encephalomyelitis. Mult Scler (1999) 5(1):2–9. 10.1191/135245899701564308 10096096

[14] Bynoe MS Evans JT Viret C Janeway CA Jr. Epicutaneous immunization with autoantigenic peptides induces T suppressor cells that prevent experimental allergic encephalomyelitis. Immunity (2003) 19(3):317–28. 10.1016/S1074-7613(03)00239-5 14499108

[15] Peron JP Yang K Chen ML Brandao WN Basso AS Commodaro AG Oral tolerance reduces Th17 cells as well as the overall inflammation in the central nervous system of EAE mice. J Neuroimmunol (2010) 227(1–2):10–7. 10.1016/j.jneuroim.2010.06.002 20580440

[16] Szczepanik M Majewska-Szczepanik M. Transdermal immunotherapy: past, present and future. Pharmacol Rep (2016) 68(4):773–81. 10.1016/j.pharep.2016.04.004 27236747

[17] Szczepanik M. Mechanisms of immunological tolerance to the antigens of the central nervous system. Skin-induced tolerance as a new therapeutic concept. J Physiol Pharmacol (2011) 62(2):159–65. 21673363

[18] Szczepanik M Tutaj M Bryniarski K Dittel BN. Epicutaneously induced TGF-beta-dependent tolerance inhibits experimental autoimmune encephalomyelitis. J Neuroimmunol (2005) 164(1–2):105–14. 10.1016/j.jneuroim.2005.04.007 15899524

[19] Majewska M Zajac K Srebro Z Sura P Ksiazek L Zemelka M Epicutaneous immunization with myelin basic protein protects from the experimental autoimmune encephalomyelitis. Pharmacol Rep (2007) 59(1):74–9. 17377209

[20] Tutaj M Szczepanik M. Epicutaneous (EC) immunization with myelin basic protein (MBP) induces TCRalphabeta+ CD4+ CD8+ double positive suppressor cells that protect from experimental autoimmune encephalomyelitis (EAE). J Autoimmun (2007) 28(4):208–15. 10.1016/j.jaut.2007.02.017 17442539

[21] Walczak A Siger M Ciach A Szczepanik M Selmaj K. Transdermal application of myelin peptides in multiple sclerosis treatment. JAMA Neurol (2013) 70(9):1105–9. 10.1001/jamaneurol.2013.3022 23817921

[22] Marcinska K Majewska-Szczepanik M Lazar A Kowalczyk P Biala D Wozniak D Epicutaneous (EC) immunization with type II collagen (COLL II) induces CD4(+) CD8(+) T suppressor cells that protect from collagen-induced arthritis (CIA). Pharmacol Rep (2016) 68(2):483–9. 10.1016/j.pharep.2015.11.004 26922557

[23] Majewska-Szczepanik M Goralska M Marcinska K Zemelka-Wiacek M Strzepa A Dorozynska I Epicutaneous immunization with protein antigen TNP-Ig alleviates TNBS-induced colitis in mice. Pharmacol Rep (2012) 64(6):1497–504. 10.1016/S1734-1140(12)70947-7 23406760

[24] Adorini L Giarratana N Penna G. Pharmacological induction of tolerogenic dendritic cells and regulatory T cells. Semin Immunol (2004) 16(2):127–34. 10.1016/j.smim.2003.12.008 15036236

[25] Hackstein H Thomson AW. Dendritic cells: emerging pharmacological targets of immunosuppressive drugs. Nat Rev Immunol (2004) 4(1):24–34. 10.1038/nri1256 14704765

[26] Takahashi K Nakayama Y Horiuchi H Ohta T Komoriya K Ohmori H Human neutrophils express messenger RNA of vitamin D receptor and respond to 1alpha,25-dihydroxyvitamin D3. Immunopharmacol Immunotoxicol (2002) 24(3):335–47. 10.1081/IPH-120014721 12375732

[27] Baeke F Takiishi T Korf H Gysemans C Mathieu C. Vitamin D: modulator of the immune system. Curr Opin Pharmacol (2010) 10(4):482–96. 10.1016/j.coph.2010.04.001 20427238

[28] Penna G Adorini L. 1 Alpha,25-dihydroxyvitamin D3 inhibits differentiation, maturation, activation, and survival of dendritic cells leading to impaired alloreactive T cell activation. J Immunol (2000) 164(5):2405–11. 10.4049/jimmunol.164.5.2405 10679076

[29] Chambers ES Hawrylowicz CM. The impact of vitamin D on regulatory T cells. Curr Allergy Asthma Rep (2011) 11(1):29–36. 10.1007/s11882-010-0161-8 21104171

[30] Gu SG Wang CJ Zhao G Li GY. Role of vitamin D in regulating the neural stem cells of mouse model with multiple sclerosis. Eur Rev Med Pharmacol Sci (2015) 19(21):4004–11. 26592821

[31] Jiao KP Li SM Lv WY Jv ML He HY. Vitamin D3 repressed astrocyte activation following lipopolysaccharide stimulation in vitro and in neonatal rats. Neuroreport (2017) 28(9):492–7. 10.1097/WNR.0000000000000782 28430709

[32] Boontanrart M Hall SD Spanier JA Hayes CE Olson JK. Vitamin D3 alters microglia immune activation by an IL-10 dependent SOCS3 mechanism. J Neuroimmunol (2016) 292:126–36. 10.1016/j.jneuroim.2016.01.015 26943970

[33] Larsson P Mattsson L Klareskog L Johnsson C. A vitamin D analogue (MC 1288) has immunomodulatory properties and suppresses collagen-induced arthritis (CIA) without causing hypercalcaemia. Clin Exp Immunol (1998) 114(2):277–83. 10.1046/j.1365-2249.1998.00706.x 9822288 PMC1905103

[34] Sochorova K Budinsky V Rozkova D Tobiasova Z Dusilova-Sulkova S Spisek R Paricalcitol (19-nor-1,25-dihydroxyvitamin D2) and calcitriol (1,25-dihydroxyvitamin D3) exert potent immunomodulatory effects on dendritic cells and inhibit induction of antigen-specific T cells. Clin Immunol (2009) 133(1):69–77. 10.1016/j.clim.2009.06.011 19660988

[35] Gonzalez-Mateo GT Fernandez-Millara V Bellon T Liappas G Ruiz-Ortega M Lopez-Cabrera M Paricalcitol reduces peritoneal fibrosis in mice through the activation of regulatory T cells and reduction in IL-17 production. PLoS One (2014) 9(10):e108477. 10.1371/journal.pone.0108477 25279459 PMC4184804

[36] Tan X Wen X Liu Y. Paricalcitol inhibits renal inflammation by promoting vitamin D receptor-mediated sequestration of NF-kappaB signaling. J Am Soc Nephrol (2008) 19(9):1741–52. 10.1681/ASN.2007060666 18525004 PMC2518439

[37] Chiuso-Minicucci F Ishikawa LL Mimura LA Fraga-Silva TF Franca TG Zorzella-Pezavento SF Treatment with vitamin D/MOG association suppresses experimental autoimmune encephalomyelitis. PLoS One (2015) 10(5):e0125836. 10.1371/journal.pone.0125836 25965341 PMC4428830

[38] Zorzella-Pezavento SFG Chiuso-Minicucci F França TGD Ishikawa LLW da Rosa LC Colavite PM pVAXhsp65 vaccination primes for high IL-10 production and decreases experimental encephalomyelitis severity. J Immunol Res (2017) 2017:6257958. 10.1155/2017/6257958 28321419 PMC5339488

[39] Blasi E Barluzzi R Bocchini V Mazzolla R Bistoni F. Immortalization of murine microglial cells by a v-raf/v-myc carrying retrovirus. J Neuroimmunol (1990) 27(2–3):229–37. 10.1016/0165-5728(90)90073-V 2110186

[40] Dulla YA Kurauchi Y Hisatsune A Seki T Shudo K Katsuki H. Regulatory mechanisms of vitamin D3 on production of nitric oxide and pro-inflammatory cytokines in microglial BV-2 cells. Neurochem Res (2016) 41(11):2848–58. 10.1007/s11064-016-2000-3 27401255

[41] Green LC Tannenbaum SR Goldman P. Nitrate synthesis in the germfree and conventional rat. Science (1981) 212(4490):56–8. 10.1126/science.6451927 6451927

[42] Fraga-Silva TFC Mimura LAN Zorzella-Pezavento SFG Ishikawa LLW França TGD Thomé R Tolerogenic vaccination with MOG/VitD overcomes aggravating effect of *C. albicans* in experimental encephalomyelitis. CNS Neurosci Ther (2016) 22(10):807–16. 10.1111/cns.12572 27321391 PMC6492784

[43] Christy AL Walker ME Hessner MJ Brown MA. Mast cell activation and neutrophil recruitment promotes early and robust inflammation in the meninges in EAE. J Autoimmun (2013) 42:50–61. 10.1016/j.jaut.2012.11.003 23267561

[44] Mimura LA Chiuso-Minicucci F Fraga-Silva TF Zorzella-Pezavento SF Franca TG Ishikawa LL Association of myelin peptide with vitamin D prevents autoimmune encephalomyelitis development. Neuroscience (2016) 317:130–40. 10.1016/j.neuroscience.2015.12.053 26762804

[45] Soellner IA Rabe J Mauri V Kaufmann J Addicks K Kuerten S. Differential aspects of immune cell infiltration and neurodegeneration in acute and relapse experimental autoimmune encephalomyelitis. Clin Immunol (2013) 149(3):519–29. 10.1016/j.clim.2013.10.011 24239839

[46] Farias AS Spagnol GS Bordeaux-Rego P Oliveira CO Fontana AG de Paula RF Vitamin D3 induces IDO+ tolerogenic DCs and enhances Treg, reducing the severity of EAE. CNS Neurosci Ther (2013) 19(4):269–77. 10.1111/cns.12071 23521914 PMC6493393

[47] O’Connor RA Anderton SM. Foxp3+ regulatory T cells in the control of experimental CNS autoimmune disease. J Neuroimmunol (2008) 193(1–2):1–11. 10.1016/j.jneuroim.2007.11.016 18077005

[48] Zozulya AL Wiendl H The role of regulatory T cells in multiple sclerosis. Nat Clin Pract Neurol (2008) 4(7):384–98. 10.1038/ncpneuro0832 18578001

[49] Venken K Hellings N Liblau R Stinissen P Disturbed regulatory T cell homeostasis in multiple sclerosis. Trends Mol Med (2010) 16(2):58–68. 10.1016/j.molmed.2009.12.003 20159585

[50] Bakdash G Schneider LP van Capel TM Kapsenberg ML Teunissen MB de Jong EC. Intradermal application of vitamin D3 increases migration of CD14+ dermal dendritic cells and promotes the development of Foxp3+ regulatory T cells. Hum Vaccin Immunother (2013) 9(2):250–8. 10.4161/hv.22918 23291929 PMC3859743

[51] Ghoreishi M Bach P Obst J Komba M Fleet JC Dutz JP. Expansion of antigen-specific regulatory T cells with the topical vitamin d analog calcipotriol. J Immunol (2009) 182(10):6071–8. 10.4049/jimmunol.0804064 19414758 PMC4572475

[52] Gregori S Giarratana N Smiroldo S Uskokovic M Adorini L. A 1alpha,25-dihydroxyvitamin D(3) analog enhances regulatory T-cells and arrests autoimmune diabetes in NOD mice. Diabetes (2002) 51(5):1367–74. 10.2337/diabetes.51.5.1367 11978632

[53] Hubert P Jacobs N Caberg JH Boniver J Delvenne P. The cross-talk between dendritic and regulatory T cells: good or evil? J Leukoc Biol (2007) 82(4):781–94. 10.1189/jlb.1106694 17652446

[54] Ray A Basu S Miller NM Chan AM Dittel BN. An increase in tolerogenic dendritic cell and natural regulatory T cell numbers during experimental autoimmune encephalomyelitis in Rras-/- mice results in attenuated disease. J Immunol (2014) 192(11):5109–17. 10.4049/jimmunol.1302254 24771856 PMC4041102

[55] Gorman S Judge MA Hart PH. Topical 1,25-dihydroxyvitamin D3 subverts the priming ability of draining lymph node dendritic cells. Immunology (2010) 131(3):415–25. 10.1111/j.1365-2567.2010.03315.x 20561084 PMC2996562

[56] Brinkmann V Davis MD Heise CE Albert R Cottens S Hof R The immune modulator FTY720 targets sphingosine 1-phosphate receptors. J Biol Chem (2002) 277(24):21453–7. 10.1074/jbc.C200176200 11967257

[57] Grishkan IV Fairchild AN Calabresi PA Gocke AR. 1,25-Dihydroxyvitamin D3 selectively and reversibly impairs T helper-cell CNS localization. Proc Natl Acad Sci U S A (2013) 110(52):21101–6. 10.1073/pnas.1306072110 24324134 PMC3876241

[58] Turley DM Miller SD. Prospects for antigen-specific tolerance based therapies for the treatment of multiple sclerosis. Results Probl Cell Differ (2010) 51:217–35. 10.1007/400_2008_13 19130025 PMC2901403

[59] Kleinewietfeld M Hafler DA. Regulatory T cells in autoimmune neuroinflammation. Immunol Rev (2014) 259(1):231–44. 10.1111/imr.12169 24712469 PMC3990868

[60] Volpe E Battistini L Borsellino G. Advances in T helper 17 cell biology: pathogenic role and potential therapy in multiple sclerosis. Mediators Inflamm (2015) 2015:475158. 10.1155/2015/475158 26770017 PMC4685148

[61] Kalueff AV Minasyan A Keisala T Kuuslahti M Miettinen S Tuohimaa P. The vitamin D neuroendocrine system as a target for novel neurotropic drugs. CNS Neurol Disord Drug Targets (2006) 5(3):363–71. 10.2174/187152706784111506 16787236

[62] Cui X Gooch H Petty A McGrath JJ Eyles D. Vitamin D and the brain: genomic and non-genomic actions. Mol Cell Endocrinol (2017) 453:131–43. 10.1016/j.mce.2017.05.035 28579120

[63] Murphy AC Lalor SJ Lynch MA Mills KH. Infiltration of Th1 and Th17 cells and activation of microglia in the CNS during the course of experimental autoimmune encephalomyelitis. Brain Behav Immun (2010) 24(4):641–51. 10.1016/j.bbi.2010.01.014 20138983

[64] Almolda B Gonzalez B Castellano B. Antigen presentation in EAE: role of microglia, macrophages and dendritic cells. Front Biosci (2011) 16:1157–71. 10.2741/3781 21196224

[65] Mancera P Wappenhans B Cordobilla B Virgili N Pugliese M Rueda F Natural docosahexaenoic acid in the triglyceride form attenuates in vitro microglial activation and ameliorates autoimmune encephalomyelitis in mice. Nutrients (2017) 9(7):681. 10.3390/nu9070681 28665331 PMC5537796

[66] Neil S Huh J Baronas V Li X McFarland HF Cherukuri M Oral administration of the nitroxide radical TEMPOL exhibits immunomodulatory and therapeutic properties in multiple sclerosis models. Brain Behav Immun (2017) 62:332–43. 10.1016/j.bbi.2017.02.018 28238951 PMC5496657

[67] Schachtele SJ Hu S Sheng WS Mutnal MB Lokensgard JR. Glial cells suppress postencephalitic CD8+ T lymphocytes through PD-L1. Glia (2014) 62(10):1582–94. 10.1002/glia.22701 24890099 PMC4141010

[68] Hu J He H Yang Z Zhu G Kang L Jing X Programmed death ligand-1 on microglia regulates Th1 differentiation via nitric oxide in experimental autoimmune encephalomyelitis. Neurosci Bull (2016) 32:70–82. 10.1007/s12264-015-0010-9 26769487 PMC5563746

[69] Dai S Jia R Zhang X Fang Q Huang L. The PD-1/PD-Ls pathway and autoimmune diseases. Cell Immunol (2014) 290(1):72–9. 10.1016/j.cellimm.2014.05.006 24908630

[70] Constantinescu CS Farooqi N O’Brien K Gran B Experimental autoimmune encephalomyelitis (EAE) as a model for multiple sclerosis (MS). Br J Pharmacol (2011) 164(4):1079–106. 10.1111/j.1476-5381.2011.01302.x 21371012 PMC3229753

[71] Dendrou CA Fugger L Friese MA Immunopathology of multiple sclerosis. Nat Rev Immunol (2015) 15(9):545–58. 10.1038/nri3871 26250739

[72] Kim EY Moudgil KD. Immunomodulation of autoimmune arthritis by pro-inflammatory cytokines. Cytokine (2017) 98:87–96. 10.1016/j.cyto.2017.04.012 28438552 PMC5581685

